# Selections from the Foreign Periodicals

**Published:** 1847-07-01

**Authors:** 


### Periscope;

OR,

### CIRCUMSPECTIVE REVIEW.

---

### Selections from the Foreign Periodicals.

---

#### On Letting Blood from the Jugular in the Diseases of Children. By C. Hildreth, M.D. Zansville, Ohio.

Dr. Hildreth, after observing that leeches, which are usually recommended in the acute affections of infancy, are sometimes not obtainable without great difficulty, goes on to state his opinion, that general bleeding in many of these is far preferable, and that the jugular vein is the one best adapted for the purpose at this period of life. "I would make this operation the rule instead of the exception in many of the acute affections of children under two or three years of age; and also in older subjects, in certain diseases of the brain and trachea. As the head in infancy is larger in proportion to the size of the body than in the adult, so also is the cerebral vascular system more developed, and hence we find the jugulars relatively larger and more prominent in the first years of life." The veins of the arm at this early age are found with great difficulty, and a sufficiency of blood hard to be obtained from them, while the near proximity of an artery has prevented practitioners opening a vein in cases where the loss of blood was urgently called for.

Dr. Hildreth considers the fear of air entering the vein as quite visionary, providing the operation has been properly performed; and believes this mode of abstracting blood as especially efficacious in inflammation of the larynx and trachea, cerebral inflammations, and congestion causing convulsions. "We also much prefer letting blood from the jugular in the acute inflammations of the thoracic viscera. One of its chief advantages is the great rapidity with which blood flows from a free orifice in the vein. A decided impression can thus be made upon the system in a very few moments, and *with much less loss of blood than would be required to produce the same effect* from a vein in the arm, or by the still slower process of leeching or cupping."

The following are Dr. H's directions for performing the operation in a more simplified manner than is usually recommended. "It is essentially necessary in letting blood from the jugular of a refractory child, *that the head and chest should be immovably fixed*, otherwise the flow of blood will be interrupted or stopped entirely. The nurse having exposed the right shoulder of the chlld, seats herself upon a low chair, and in holding the child across her knees carefully confines its arms. The surgeon, seated at her side, receives and secures the child's head between his knees. With the thumb of the left hand, he now compresses the jugular across where it crosses the first rib, while the remaining portion of the same hand is emloyed in fixing the chest of the child against the person of the nurse. The right hand of the operator being at liberty, he makes the opening into the vein. The blood is received in a cup, the edge of which applied a little below the orifice, likewise serves to compress the vein. From a robust child blood escapes with astonishing rapidity, particularly when he cries or struggles. No effort, therefore, should be made to keep him quiet, his cries being rather encouraged as they expedite very much the operation. A sufficient quantity of blood is very soon lost, the colour of the lips and cheek of the child telling when syncope approaches much more certainly than the pulse. The quantity of blood desired having been lost, a compress is applied to the orifice, and the pressure taken off from the vein below. After the child becomes quiet, the compress is removed, and the wound closed by a piece of court or adhesive plaster. This is much the best dressing, the compress and bandage usually advised being very objectionable. It not only obstructs the free return of blood from the head, but its presence irritates the patient, and if not well adjusted may promote the flow of blood or interfere with respiration. If, however, all pressure be removed from the vein below, blood will not escape if no dressing be applied, except the child cry or struggle. We therefore much prefer, if the proper dressing be not convenient, leaving the orifice uncovered, and directing the nurse to apply the compress for a moment, should blood escape during the cries of the child."

However young the child may be, Dr. Hildreth feels convinced that, in the above-named inflammatory diseases (he prefers leeches in inflammations of mucous membranes in general and of the abdominal viscera in particular) accompanied with high fever, a smaller quantity of blood so abstracted will suffice than if it had been taken in the ordinary manner—and in young children the jugular is the only vein of sufficient size to admit of the certain and safe abstraction of blood. He relates a few cases in proof of the prompt benefit occurring from the operation."—*American Journal Med. Sciences,* No. 26, pp. 369, 74.

[We feel great pleasure in giving an abstract of the above paper, as we feel convinced it draws attention to an important practical fact. We are no advocates for depletion of children to the extent sometimes recommended; but in cases wherein the prompt loss of a certain amount of blood seems indicated, we agree with Dr. Hildreth, that it may oftentimes be more advantageously abstracted by jugularotomy than by leeches. We shall not soon forget the instant and marked relief we once saw thus produced in a case of croup.—*Rev.*]

---

### Observations Introductory to a Course of Clinical Surgery. By M. Malgaigne.

From the time of Celsus certain physical qualities have been expected of the surgeon, namely, that he should be young, adroit, and ambi-dextrous. M. Mayor of Lausanne would send him to study among the carpenters and joiners; in a word, his has been looked upon as a mere mechanical art, *quod in therapeiâ mecanicum,* according to the expression of Richerand. But is this the only employment of the surgeon? Is it as in the middle ages, when the physician sent for him to perform certain mechanical operations and then dismissed him? No! After having been so long a subaltern, the surgeon must now take that rank which is his due. The physician may be ignorant of surgery, but the surgeon should know medicine; for in how many instances are surgical affections preceded or followed by phenomena which involve the entire economy! That he should be adroit can but be of great utility to him; but as to being ambi-dexter, few persons can boast of this, and frequently those who can, as Rivarol observes, have only two left hands.

For our parts, what we demand of the surgeon before all things is, that he have a healthy, cultivated, and philosophical mind, and a high moral character, and that he should not be, as Celsus would have him, without pity. Surgical morality is of especial importance in respect to new operations—experiments to be tried on the living man. We must think more than once when human life is at issue. He should possess a philosophic spirit, in order that he may verify before he believes, not accepting the dictums of his teacher without examination. Faith in authority at the present day must yield to faith in facts. His mind should be of a logical cast, so that he may not mistake analogies for proofs, or draw too hasty conclusions, such as inferring, for example, from the phenomena observed in frogs, the certainty of similar facts in man.

From the time of J. L. Petit, surgery and medicine have been based upon normal and pathological anatomy. This is an error. Normal anatomy is truly a great aid, for it teaches you the position of the nerves and blood-vessels, and of all the internal organs. It is essential for the mechanical part of surgery or operative medicine. But has not surgery just as much need of cutlery and pharmacy? and should we therefore not accord them as important a position as anatomy? The true basis of surgery is *Physiology;* but not the physiology which has been up to the present time almost exclusively taught you, and which has been termed the " Romance of Medicine." Of what use in practice is such physiological knowledge? How are your therapeutical procedures influenced by knowing that it is the heart which propels the blood, the lungs which respire, the liver which secretes the bile, or the kidneys the urine? Even the discovery of the circulation has not advanced the treatment of disease a step. Before Harvey's time Botal bled *coup sur coup*, and while recognizing what there is of beautiful in this discovery (for the truth is always beautiful), we may almost say that, from that epoch, therapeutics, as regards blood-letting, have pursued a retrograde course. In fact, experience seems to have led the ancients to bleed from a variety of veins; and upon what grounds is this practice abandoned?

True medical and surgical physiology, that which employs itself in discovering the manner in which Nature repairs the breaches made in the human body, dates from JOHN HUNTER. He in fact first investigated the manner in which she comports herself in the healthy man for the resistance to causes of disturbance or ruin. Physiology, so understood, forms the basis of the rational part of surgery. It will direct us to the study in its origin, its causes, its progress, and its consequences, of that great phenomenon, inflammation. It will shew us how to direct or repress, or to excite it, as the case may be. Reflect upon the import of this change of basis—physiology in the place of anatomy—and you perceive in it consists an entire revolution in the art of surgery.—

There is something else besides his organs which appertains to man. The organs exist in the dead body. There is besides in the living body a power whose nature is unknown, a power which circulates the blood, generates motion and thought, and which offers resistance when an external agent deranges that order which constitutes health. It has been differently regarded; some considering it as dependent upon physical or chemical laws, others as mechanical. But can we in this way explain even the most ordinary fact? You cut your finger; the blood flows, and then stops after the formation of a clot. Oh! it is the heart, it is said, a hydraulic machine which propels the blood until the obstacle produced by chemical decomposition closes the vessels. In nearly the same manner may the red circle surrounding the wound be explained. Exosmosis then suffices; just as endosmosis explains its disappearance. But why do not these phenomena thus come to pass in every case? Why do they occur at one period rather than another? Why do we not see them on the dead body, while it differs not as regards its chemical relations from the living man? It is because *vital force* exists no longer. What is this vital force, then? No one knows. We admit it just as we do attraction and chemical affinity. But what matters its nature? Sufficient for us to know its action. This knowledge is however of the highest import; for it is only by studying the phenomena by which it reveals itself, by penetrating the laws which govern it, that we can learn how to direct it. Let us examine well then how it acts in order to resist external agents; but, above all, let us study it in man, and preserve ourselves from the too common error, the abuse of experiments upon animals. These are useful only when they are made upon the nearest approaching species, or upon such as regards the point in question are identical. We could not, for example, describe inflammation in man according to the phenomena observed in the dog, in which there is hardly any suppuration, or the rabbit, in which it exists not at all. Let us then investigate the mode of action of the vital force, and first let us observe it under the most simple circumstances. We have received a cut; the blood flows, then stops; the capillaries are injected, and inflammation is established. If we leave Nature alone, and trust to the vital force, we observe that it is essentially a *vis medicatrix*, and that in this case it alone suffices; whence the conclusion, that in *traumatic iuflammation the surgeon's first duty is to do nothing.* Do nothing! An important dictum this that needs interpretation. Nature, at the commencement, we have said, is essentially a medicatrix, but upon the condition that her work is not interfered with. Wait to act until she wanders or deviates, and then intervene, to place her again in the right path. Watch like a vigilant sentinel, in order to remove whatever may interrupt her proceedings. To fulfil this great therapeutical indication, which of itself constitutes three-fourths of surgery, we are obliged to modify the treatment of the most obstinate as well as the most simple diseases. What has a surgeon to do in a simple fracture? Nothing. Nature alone will well repair the damage, provided she is protected from interruption during her labours. Among the most frequent obstacles is movement of the parts; and our care is limited to securing, by the best-adapted apparatus, the complete immovability of the part. As to bandages, what is their effect but to deprive the part of the vivifying action of the air, and etiolate it, even if they do not produce a local scorbutus. We may say as much of resolvents, such as white lotion, spirits of camphor, &c. What is there to resolve in a fracture? The swelling which accompanies the inflammation? Is it not necessary for the consolidation. The effused blood? Nature will remove that without your aid. If there is displacement of the fragments, the hand of the surgeon is required; and this is mechanical, or, if you like, operative medicine. Restore the form of the limb, fix it conveniently, and retire. Let Nature resume her place, and do not say that you have effected a cure. You have reduced the fracture; the cure comes from Nature alone.

Simple surveillance is also our essential and only duty in all those simple inflammations, which in all their symptoms and progress are analogous to traumatic inflammation, and which come on from unknown causes, or under the problematical influence of cold, draughts of air, &c. The same principles will apply, *e. g.* to white swellings, diseases whose cause is often traumatic, and which continue long in the condition of simple inflammation before acquiring the gravity which subsequently characterizes them. At the commencement, and indeed as long as the disease has not passed beyond the limits of simple inflammation, prevent the movement of the part, that so frequent obstacle to the efforts of Nature. Remove all causes of pain, and especially such as arise from faulty position, of such common occurrence when the disease has been of long continuance; and discard all that perturbatory medicine which only can make use of leeches, blisters and moxas. Of these means, the employment of which now seem the general rule, some may be useful in rare and special cases; but what shall we say to a blister put on for the relief of pain which only depended upon faulty position! The common result of such therapeutic procedure is to convert an ordinary synovitis into a fuugous tumour, and to give the surgeon occasion to shew his dexterity in depriving the patient of a limb, which, by proper care, he might have preserved for him.

The considerations we have entered into give rise to new views, which may serve to establish the superiority of surgery over medicine. The facility with which we may ensure immovability to a limb, and the impossibility of procuring the inactivity of the heart, lungs, &c. explain why surgical inflammations are generally so much easier of cure than medical ones. In conclusion, expectation in surgery cannot be too highly recommended; but an intelligent cxpectation. Nature is not always a *vis medicatrix*, and then it will not do to remain a simple spectator. Direct the vital force, govern it, resist it if necessary by the most energetic surgical means; and even in certain desperate cases abandon it to itself. Resume your passive character, the power of diagnosis and prognosis alone remaining to you. It is better to deplore a case as desperate, and to let the patient die at least naturally, than hasten his end by an operation that nothing can justify.—*Gazette des Hôpitaux,* No. 57.

---

### Treatment of Epilepsy.

While detailing some unsuccessful experiments he had been making upon the employment of Ether in epilepsy, M. Moreau delivered the following important observations:—" Every medicinal substance has in turn failed when employed against this terrible neurosis, as everybody knows. But what every one does not so well know is, that all, or almost all, these remedies succeed during the first periods of their employment. That is to say, the attacks diminish, or cease entirely, and even suddenly. But just in proportion to the extent of the apparent amelioration, the more the attacks seem to have yielded, the more terrible do they become when they are reproduced. They may then even place life itself in danger, which they never did before. This fact is of great practical importance, and I have seen hundreds of examples of it during the now near seven years that I have sought, by all the remedies proposed at home or abroad, and some of my own devising, to combat this dreadful disease.—*Gazette des Hôpitaux,* No. 38.

---

### Treatment of Burns.

M. Jobert and M. Malgaigne pursue very different modes of treating Burns at the St. Louis, where a great number of these cases are admitted. M. Jobert employs *refrigerants,* laying bladders of ice upon the parts, previously covered with simple dressing. From this treatment he states that he obtains, 1, prompt cessation of pain : 2, an abolition or diminution of inflammatory action : 3, a diminution of the suppuration which precedes or follows the separation of the eschars; and 4, a more rapid and prompt cicatrization; but it is, however, contra-indicated when thoracic complications exist, or when a predisposition to these is present; or when the burn, situated in the chest or back is very extensive, M. Malgaigne prefers the oleo-calcarous liniment and carded cotton; associating the two because the cotton alone does not so rapidly relieve the pain, and the liniment alone does not protect the surface from friction. Like M. Velpeau, he prefers equal parts of lime-water and olive oil; but M. Miquel recommends 2 parts of the former to 1 of the oil. This produces a speedy cessation of the pain.—*Gazette des Hôpitaux,* No. 59.

[We have employed with much advantage the application recommended by Mr. Bulling, viz., covering the parts with lint soaked in a mixture of 1 part treacle, 3 water—using it at a temperature of 98°.—*Rev.*]

---

### Ectrotic Treatment of Small-Pox.

Dr. S. Jackson states that he has found the free application of the Tincture of Iodine by means of a sponge, causes an abortion of the eruption, and prevents pitting or the disagreeable discolouration which usually follows the disease.—*Philadelphia Medical Examiner.*

### Notes of Cases of Chronic Diffused Arteritis, cured by the Antiphlogistic Method after the Failure of different Modes of Treatment in the hands of others. By Dr. Rinino of Turin.

The greater importance most physicians attach to mere symptomatic appearances than to pathological conditions in the diagnosis and treatment of diseases, especially those of a slow and chronic nature, is the frequent cause of the most pernicious errors, into which even practitioners otherwise of great excellence fall; errors which have often led patients to certain death, who, treated conformably to the maxims of modern experience (based upon facts examined by the light of a philosophical pathology and guided by pathological anatomy), might have recovered their lost health, and lived for many years. Such errors are more easily committed in those cases in which certain chronic diseases are marked by nervous symptoms, as in the case of hysteria, hypochondriasis, believed by many practitioners to be dependent upon a totally different state to that of phlogosis. Add to this that, when treating patients of emaciated or cachectic habits, the subjects of hæmorrhage or discharges, women, children and old persons, ill-fed individuals, such as are subjected to prolonged mental employment or abuses of any kind, we are led to suspect their complaints are of an asthenic rather than of an inflammatory nature. And yet, if the symptoms be accurately examined, the indications of slow inflammation will be generally detected; whence it follows that the treatment pursued in these emergencies may be actually injurious, or, if too bland and palliative, will allow the patient to die from the degeneration of tissue and other consequences unsubdued inflammatory action produces. What is true in a general way in regard to morbid conditions dependent upon slow inflammation mistaken and badly treated by practitioners, applies to *chronic diffused arteritis,* of which I now publish a few of the more important cases that have come under my notice during a short space of time.

The *first* of these occurred in the person of a lady, who, having arrived at the time of life when the menses were leaving her, was tormented at the periods by various vague pains in different parts of her body, and vibratory pulsation at the præcordium, the neck and temples. Sleepless nights, bad digestion, and confirmed melancholy existed. Two practitioners, consulted in succession, observing the feeble state of the patient and the absence of fever, pronounced her ailments of a nervous and asthenic character, and prescribed the usual remedies for these conditions. Dr. Rinino was now called in, and, finding the pulse frequent, " metallic," and vibratory, he pronounced the case one of arteritis, notwithstanding the absence of fever. Ten bleedings were practised in the course of a fortnight, leeches and then ice applied, a rigorous diet adhered to, and various contra-stimulants prescribed, and the lady entirely recovered her health, which she retained by taking the precaution of losing blood two or three times a year.

*Case* 2.—Signor Panizza had suffered for some years under the symptoms of hypochondriasis. Indigestion, epigastric pain, costiveness, emaciation, insomnia, a chlorotic aspect, and confirmed melancholy were among the symptoms which induced his attendants to consider his case one of asthenic hypochondriasis, and to treat it unavailingly by nutritious diet, pharmaceutical stimuli, and mental distractions. Afterwards, some congestion of the liver being suspected, leeches to the anus and purgatives were prescribed. By the first plan of treatment all his symptoms were aggravated, by the latter only temporarily relieved, and the patient seemed fast sinking into a hopeless state when the author was called in. Notwithstanding the feeble condition of the patient, he declared a chronic arteritis to be the cause of his sufferings, and prescribed free depletion for its removal. He based his diagnosis upon a constant arterial vibration felt and heard at the epigastrium, and upon the sharp, metallic character of the pulse—the patient being however apyretic. Nine bleedings and a leeching rapidly followed each other, a rigorous diet and contra-stimulant remedies being simultaneously employed, and in forty-five days the patient was convalescent.

*Case* 3.—Signor Sasso (ætat. 60) was seized, in 1845, with a complicated condition of ill health, the chief feature of which was diagnosticated by his attendants as ischiatic neurosis and treated with palliatives, without effect, until at last the patient seemed to be in a state of great danger. He was of a very sanguine temperament and weak constitution, accustomed to good living and little exercise. He complained exceedingly of an epigastric pulsation which tormented him whatever position he assumed, and, although he had no fever, he presented the rapid, metallic, vibratory pulse observable in chronic arteritis. He suffered acute pain in the hip-joint, which the slightest movement exasperated. Nine successive bleedings and a free leeching with a spare diet were resorted to, and the various contra-stimuli, especially kermes, extract of aconite, and secale cornutum, alternating with purgatives, were prescribed. During a month convalescence was satisfactory: insomnia was exchanged for refreshing rest, and the supposed sciatica, which the author believes to have been an arthritis, gradually disappeared.

*Case* 4.—Occurred in the person of a girl of about four years of age, who for two months had had the eyes closed in consequence of an obstinate psorophthalmia, the supposed residuum of a crusta lactea, and against which various demulcent remedies had been in vain employed. Dr. R. was called in in consequence of a profuse epistaxis, and was struck with the marked arterial vibration in spite of the loss of blood, which amounted to almost two pounds (Austrian). He informed the parents that the epistaxis was in fact a fortunate occurrence, and eventually persuaded them to allow him to abstract yet more blood (which was cupped and buffed), on three separate occasions. Having thus, in some degree, quieted the arterial vibration, he prescribed kermes in union with the extract of aconite and Dover's powder; and in a fortnight the child was able to open her eyes, the psorophthalmia and arteritis gradually yielding to a persistence in the medicines.

*Case* 5.—No one, says the author, accustomed to the examination of these cases could be deceived in diagnosing chronic arteritis as the cause of the serious and prolonged illness of Signora Pinta. Her countenance was the colour of virgin wax; vigorous pulsations prevailed at the præcordium, accompanied by a sense of weight and anxiety: the cardiac pulsations imparted a very vibratory sensation to the ear, and the pulse was strong and metallic. The arteries of the neck and temples also pulsated rigorously. There was no fever. A sense of universal uneasiness tormented the patient, and in turns violent pains attacked the abdomen and head. She was absolutely sleepless, had become much emaciated, and laboured under an inexpressible sense of prostration of strength. Her illness dated four months back, when Dr. R. saw her, and at first putting on a febrile aspect, her attendant had bled her four times with temporary relief, and other four times after she had again relapsed. Other assistance being sought, her yellowish aspect and wasting away, and the duration of her illness led to the belief of the existence of a passive congestion of the liver, dependent upon an asthenic diathesis. After leeching the anus, stimulating remedies were employed with the effect of plunging the patient in a worse state than ever. Our author, called in, quickly corrected the error of diagnosis, and proceeding more actively in the same course of the more timid practitioner first consulted, in a very few days had bled the patient thirteen times to the extent of a pound, besides leeching her. In a month she had entirely recovered.—*Annali Universali*, Vol. cxviii. 29.

[*Other cases are adduced, but these are the principal ones, and considering that whenever Dr. Rinino specifies the amount of blood taken, he states this to have been a pound, and that his bleedings were sometimes repeated five, ten, or even thirteen times, and that for not very well characterized chronic inflammation, he proves himself to be a worthier follower in the footsteps of Guy Patin than is often met with in these degenerate times. The lavish use of the lancet in fact prevails, as we have observed in our notice upon Tommasini (p. 271), to a greater extent in Italy than in any other European country; but we imagine that, even there, Dr. R. will make few proselytes. That cases may every now and then be met with which may be benefited by such treatment we do not doubt, and the placing them on record has its utility by exciting attention to the important fact indicated by the author, and certainly too frequently overlooked, that the essential inflammatory character of a disease may be masked or concealed by a train of pseudo-asthenic and nervous symptoms, which have supervened. Infinite mischief would however result, if this exceptional case were generalized into a principle.]—*Rev.*

---

### On Intercostal Neuritis and Neuralgia. By Dr. Beau.

Since the researches of MM. Bassereau and Valleix *intercostal neuralgia* has taken its place among the acknowledged affections of the economy; but with it other cases, for which the term *neuritis* would be more applicable, have been confounded. Dr. Beau's attention was first directed to the subject while contemplating the nature of the painful sensations in injuries of the ribs. Of two such cases, in the one case a severe contusion of the thorax, and in the other actual fracture, took place at the junction of the posterior and middle thirds of the ribs; and in both cases, while some degree of pain existed at the precise seat of injury, that of an intense character was located anteriorly near the sternum. It was the latter that became intolerably increased by coughing, sneezing, or other respiratory efforts. In these cases the pain was explicable only on the supposition of an inflamed state of the intercostal nerve consequent upon the injuries, the severest suffering being referred to the periphery in consonance with a well-known law.

Neither of these patients dying the positive proof of the existence of such neuritis was wanting; but these cases led to the consideration of others of much more common occurrence, in which the existence of the peripheric pain and the means of proving its dependence upon an inflamed state of the nerve alike exist. Such are cases of *inflammation of the pleura, whether simple or complicated with pneumonia.* It is familiarly known that the "pain in the side," so constantly present in these, is seated in the great majority of cases near the breast. It is, in fact, but the expression of pain at the peripheric extremity of the intercostal nerve, induced by inflammation of the portion of this nerve which is in contact with the inflamed pleura. The posterior portion of the nerve alone is inflamed, and yet the severe pain is excited at its periphery.

The intercostal nerves during the posterior portion of their course, that is from the articulation of the ribs to their angle, are in immediate contact, on the external side, with the external intercostal muscle, and, on the internal side, with the parietal layer of the pleura. From the angle of the ribs to their termination, the nerves cease to be in immediate relation with the pleura, being separated from it in all the rest of their course by the internal intercostal muscle. It seems scarcely possible for the nerve to be so closely in relation to the inflamed pleura

---

* For an account of similar cases by Tommasini see Med.-Chir. Rev. N. S., I., 300.

without its participating in the diseased action; and, in point of fact, at post-mortem examinations we always find this portion of the nerve more or less inflamed during the whole portion of its course that is in contact with the inflamed pleura, such inflammation not extending beyond the angle of the ribs, where the nerve becomes separated by the muscle from the pleura. There is frequently a somewhat intense injection, not only of the neurilema but of the nerve itself, with enlargement of its substance, as may be seen by comparing it with the uninflamed nerves in contact with uninflamed portions of pleura. The inflamed nerve has not seemed more friable than the others, but is sometimes slightly adherent to the contiguous pleura. It is to be remembered that pleurisies, and pleuro-pneumonias, are situated in the great majority of instances at the posterior portion of the chest, and yet the pain is felt at its anterior portion, as already observed. If this statement be correct, the pain induced at the anterior extremities of the intercostal nerves should vary in its longitudinal direction according to the height in the thorax at which the pleuritic inflammation is seated, and this is precisely what takes place; for, accordingly as the pleurisy affects the four or five first, or the four or five lower intercostal nerves, so is the pain felt at the anterior portion of the corresponding intercostal spaces. And, as the anterior extremities of the five last nerves, instead of turning up with the cartilages, proceed downwards and forwards, between the muscles of the abdominal parietes, towards the median line, the pain proceeding from the inflamed pleura is then manifested in the abdomen. It results from these details, that the seat of the peripheric pain of the inflamed nerve may serve as an excellent guide to the exact seat of the pleurisy, as all we have to do is to trace directly backwards along the course of the affected nerve. If local bleeding applied to the seat of pain instead of the seat of the neuritis, readily dissipates the pains, it does so because it operates a derivation at a certain distance from the inflamed part upon the intercostal vessels feeding the inflammation—just as, in orchitis, we place leeches over the cord, and not upon the scrotum.

Ordinarily all the nerves in contact with the inflamed pleura are equally inflamed, but all are not equally painful at their extremity. It will be found in general, that that nerve is most affected which corresponds to the rib possessed of most extensive movements. This is why, in most cases, the patients refer the most vivid pain to the anterior portion of the sixth or seventh intercostal space, because in most patients, and especially in men, the seventh rib is that which executes the greatest amount of movement. The patients will generally complain of pain at one of the intercostal spaces, but it is rare for only one nerve to be thus affected; and, if we compress the spaces adjoining that at which the sensations of the patient seem to be centered, we find that others are similarly affected, though in different degrees. The difference in the intensity of suffering is very great; for, while some nerves are excessively painful, others, equally inflamed, give signs of scarcely any pain. Differences in pathological susceptibility analogous to this are however familiar to attentive observers; and it is the entire absence of such susceptibility in certain individuals that permits *latent* pleurisy and pleuro-pneumonia to become developed without the manifestation of pain in the side, or any other symptom of the disease.

We have hitherto laid it down as a law, that the posterior inflamed portion of the nerve *only* manifests pain at its anterior extremity; but there are some exceptions to this. We have observed, in the most careful manner, cases of pleurisy, in which pain existed simultaneously at the extremities of the intercostal nerves, and at the portion of the spinal column corresponding to the affected nerves. The latter pain is not however spontaneous like the former, but for its induction requires slight pressure to be made on the side of the spinous processes corresponding to the inflamed nerves, and then as many painful points will be recognized posteriorly as anteriorly. Every one is aware that, during percussion of the posterior portion of the thorax in pleurisy, pain is produced.

This is always referred to the inflamed pleura, but in fact is a posterior radiation of the inflamed intercostals. This pain at the posterior portion of the thorax is not fixed, as the anterior pain in the intercostal branch properly so called, but in the branch which terminates in the muscles and skin of the back; and yet in necroscopies we are enabled to show that this dorsal branch is no more inflamed than is the anterior extremity of the intercostal nerve, the pain being, in the one case as in the other, a distant result of inflammation affecting the portion of nerve in contact with the inflamed pleura.

These pains of the side, then, commonly termed *pleuritic*, are justly so called, on account of their relation to pleurisy. But pleurisy does not produce them *directly*, inasmuch as they result from the inflamed state of the proximal extremity of the nerve. The pains which continue to be felt after the cessation of a pleurisy, and which are usually referred to adhesions, are, in point of fact, produced by the neuritis become chronic. When there is inflammation of the lung without inflamed pleura, we have then no pains in the side, no neuritis capable of producing them having been generated. There is another form of pleurisy, in which the intercostal nerves are liable to become inflamed—that which is consecutive to pulmonary tubercle, and which is then seated at the upper part of the chest. The pain resulting from this is felt at the anterior part of the first intercostal spaces, but is much less severe than that of acute pleurisy. Those dull pains existing just under the clavicles, and which, according to pathologists, are a frequent symptom, and an immediate result of the presence of tubercle, are, in fact, produced by the development of pleuritis consecutively to the tubercle. Besides these pains, phthisical patients occasionally suffer from others in the supra-clavicular region of a far more intense character, forcing cries from the patient, and requiring the endermic use of morphia for their relief. These, in all probability, depend upon a neuritis of the first intercostal nerve, which sends one of its branches to anastomose with the brachial plexus. This last is in communication with the cervical plexus, and we can understand how the neuritis of the first intercostal may in this way induce pain in the region of the neck; and even down the arm.

In comparing intercostal neuritis with *intercostal neuralgia*, we should first distinguish the varieties of this last. The most important of these is that described by M. Bassereau as "commonly sympathetic of an affection of some viscus, whose suffering is transmitted to the intercostal nerves by means of the anastomoses of the great splanchnic." M. Bassereau believes the uterus and its appendages to be the seat of the irritation thus propagated, inasmuch as women are much oftener the subjects of intercostal neuralgia than men, and that the women so affected, in the majority of cases, are suffering from some disturbance in the uterine functions. M. Beau demurs to this latter conclusion, believing that disorder of the digestive organs is the point of departure of the neuralgia; for—1, the great splanchnic is in communication with the semilunar ganglions and lunar plexus; 2, although these females are suffering from derangement of the uterine functions, they are so in a much more marked degree from that of the digestive organs; and, 3, that in all the male patients liable to this neuralgia, the number of whom is greater than M. Bassereau believes, there is a marked disorder of these. Dyspeptic symptoms need not be excessive, and yet the disorder they indicate may have a pathogenic influence upon various organs. So connected with dyspepsia has M. Beau long considered this neuralgia, that he always terms it in his clinical lectures the *dyspeptic neuralgia.* Wherever such neuralgia disappears completely, the digestive functions have recovered their normal integrity; and to combat the neuralgia effectually we must attack the dyspepsia—all means directed to the relief of the former, without attention to the latter, being merely temporary and palliative in their operation. This *dyspeptic neuralgia* affects principally the nerves corresponding to the ganglions, which furnish the constituent branches of the trisplanchnic nerve, that is to say, the intercostal nerves comprised between the 5th, 6th, and 7th intercostal spaces. As in neuritis, there is always one nerve more affected than the neighbouring ones, and that corresponding to the rib possessed of the most extensive movements. Generally five or six intercostal spaces are simultaneously attacked, although in different degrees. This neuralgia, as shown by M. Valleix, also frequently presents three painful points: one at the termination of the intercostal branch, another where the middle perforating branch is given off, and the third over the dorsal branch, near the spinous processes. Its duration is generally chronic, like that of the dyspepsia upon which it depends, and during its progress it exhibits sometimes regular, but generally irregular intermissions.

The second variety of intercostal neuralgia is that dependent upon rheumatism, *rheumatic neuralgia,* commonly termed *pleurodynia.* Very frequently only one of the intercostal nerves is affected, but the pain is very intense, especially if excited by pressure. It sometimes reaches the extent of preventing the patient laying down, and impeding the respiratory movements, which become short, irregular, jerking, and accompanied by interrupted exclamations. It is worse at night than by day, the maximum of its intensity being seated at the anterior portion of the intercostal nerve. It may be sometimes excited posteriorly by pressure over the dorsal branch of the nerve, but it never spontaneously arises there, as it so frequently does in neuralgia of a dyspeptic origin. This acute form only continues for some days, and may be accompanied by fever, when it puts on the greatest resemblance to neuritis. It affects men as frequently as women, while dyspeptic neuralgia, just as dyspepsia itself, most frequently affects women.

In comparing neuritis with these neuralgias, we observe that their symptoms have much resemblance, especially as regards rheumatic neuralgia. The pain of this, as of neuritis felt towards the anterior portion of the intercostal space, is very intense. It is less so in the dyspeptic variety, and the patient in the latter frequently complains of pain over the dorsal branch of the nerve, which in neuritis or rheumatic neuralgia is generally only produced upon pressure. The dyspeptic form especially affects the nerves between the 5th and 7th rib, while the seat of pain varies in the others according to that of the pleurisy, or the part affected by the cold, which has induced the rheumatism. Dyspeptic neuralgia is liable to frequent intermissions and exacerbations, which neuritis and rheumatic neurosis rarely are.

"The ideas, so long since considered as classical, respecting the vivid sensibility of the pleura and the pungent kind of pain resulting from its inflammation, ought, I believe, to be discarded, seeing that the acute and pungent pains of pleurisy do not proceed immediately from the inflamed pleura, but from the intercostal nerves, which the inflammation of the pleura has invaded."—*Archives Generales,* T. 13, pp. 161—181.

[This paper must be regarded as containing an interesting and ingenious suggestion on the part of Dr. Beau rather than a demonstration; for he particularizes no anatomical inspections that he has made in corroboration of his views. The accuracy of the conclusions it contains will however doubtless be soon sufficiently tested.—*Rev.*]

---

### On Exostoses and the Operations they require. By M. Roux.

Pathologists have comprehended under the general term Exostosis affections of an entirely different nature. In this way have been confounded together—1, Simple, chronic, partial or general hypertrophy of a bone: 2, Osteosis with circumscribed swelling of the affected bone, so often observed as a symptom of constitutional syphilis: 3, Aneurismal tumours of bone: 4, Sarcomatous swelling;

5, The degeneration termed by A. Cooper fungus or medullary exostosis: and 6, those tumours, organized like the bony tissue itself, which spring from the bone like natural apophyses, or exhibit themselves as great tubercles or excrescences within the external layers of the bone, upon which they seem as if implanted. These last are, properly speaking, exostoses, and it is to these alone attention is directed in this paper. I distinguish them carefully from those supernumerary apophyses which the bones of some subjects present, and which are almost always multiple and often very numerous in the same individual, and are more or less similar in form to the natural apophyses. The exostosis, properly so called, is, on the contrary, almost constantly solitary; an anormal condition confined to one point of the osseous system. Once, however, I saw two very compact exostoses situated on the maxillary bones, one on each side the nose; and quite recently I have seen a young man, having an exostosis on the thumb of the right hand, and another on the index finger of the right. This was the first time I had seen true exostoses, and those voluminous ones, on the phalanges.

I was sometime since about to collect my various observations upon this affection together, when the following case presented itself to my notice. A man, æt. 28, consulted me for a hard nipple-shaped tumour, firmly attached, like an appendix or apophysis, to the lower part of the femur, at the uppermost border of the ham. Although seemingly the size of a child's fist, it barely raised the skin beneath which it was placed. By careful examination, and separation of the muscles among which it was placed, a narrower base or kind of thick pedicle attaching it to the femur could be distinguished. The growth had commenced some years before, and after continuing somewhat rapidly it remained stationary for about eighteen months or two years. Quite recently, a collection of fluid had formed between the culminant portion of the tumour and the external soft parts, and this latterly has given the tumour the appearance of increasing. I did not hesitate to regard this fluid as a little synovial sac, a sort of hygroma, just like that which so frequently forms in the bursæ of the patella, olecranon, &c. under the influence of contusions or continued pressure; and my opinion was strengthened by the prompt disappearance of the fluid upon the application of a strong solution of muriate of ammonia. As the exostosis itself had been for some time stationary, caused no deformity and little inconvenience, notwithstanding that the vessels and nerve must have undergone some change of position, any operation for its removal was out of the question.

This made the seventh time that I had observed a pediculated exostosis of this kind, seated at the lower third of the femur, and constituting an isolated affection. In most of the cases the tumour was seated anteriorly, in the immediate vicinity of the capsule. Whence comes this disposition of the lower part of the femur to be especially affected? I know not: but if I may judge from three or four cases I have met with in the humerus it is the *upper* part that is most constantly affected. According to my experience, too, the different parts of the skeleton vary much as to their degree of liability to exostosis. The femur seems so far beyond any other bone: then comes the humerus, the maxillæ, and then the phalanges, especially the last phalanx of the great toe, raising the nail, distending its matrix, and inducing a more or less acute pain. Dupuytren met with these cases of exostosis of the toe several times, and his directions for their removal are excellent. The nail is to be removed after longitudinally dividing it, and the tumour, which is rarely larger than a large pea or the point of the little finger, cut away with a saw, or better still, on account of its spongy texture, with a pair of cutting forceps.

Whatever place they occupy, or upon whatever bone they become developed, these tumours have certain general characteristics which it is useful to be aware of. 1. A very remarkable one is that, although they may be found in adults, these persons were first affected by them at an earlier period of life. It is for the most part individuals from 12 to 20 years of age that consult us concerning them. 2. They do not possess an unlimited power of increase. Arrived at a certain size they cease to grow, and I would risk stating that their growth ceases with that of the bones. 3. In comparing them with each other after they have acquired all the development they are susceptible of, their size seems in relation to that of the bone affected. They are generally larger in the femur than the humerus, greater still upon the bones of the pelvis, while they continue for a very long period small at the last phalanx of the great toe. So, too, with reference to certain tumours of the soft parts, such as lipoma and encysted tumours of various kinds, their power of growth seems very much dependent upon the size of the region of the body they occupy. 4. These tumours are not united to the bone by a pedicle properly so called, but only a certain degree of narrowing of their base. The principal part of the tumour is usually irregularly spherical, having an irregular and semi-cartilaginous surface. The central part of its substance may be somewhat spongy, but the tissue becomes much more compact as we approach the narrower base. 5. The connection of these tumours with the parts that surround and cover them is various. When the exostosis is covered in part by a muscular layer, it is sometimes separated from it by a slightly condensed cellular tissue which yet allows the muscles to slide over the exostosis; while, at others, a small sac is formed containing fluid. In some cases, again, the exostosis and the muscles become firmly joined together, so as to render all motion of the latter over the former impossible; and, if an operation were undertaken upon parts so situated, the pedicle being divided without the tumour having been previously exposed, it might happen that this could not be removed without removing a portion of the substance of the muscle.

Considered as changes of organs, these osseous tumours assuredly form the simplest of diseases. They give rise to no inherent phenomenon or symptom; they have no particular tendency to degenerate; each is, as it were, as a little bone attached to the larger, and at most only susceptible of undergoing the same changes as this last. But as the exostosis approaches the surface it produces more or less deformity, and where it compresses or distends the soft parts it may give rise to more or less suffering, while situated within a cavity (*e. g.* the orbit or pelvis) it may induce most important functional disturbance or obstruction. In the limbs, the action of the muscles may be rendered painful or imperfect by them. One thing has always struck me in respect to these tumours seated beneath the muscles, namely, that the inconvenience and pain they occasion diminish, doubtless from the effect of habit, in proportion to the length of time the exostosis has existed. It is a good reason for exhorting our patients to have patience, and not yielding too readily to their desire for undergoing an operation."

M. Roux furnishes at some length the particulars of the various cases calling for operations and the mode in which he performed these. These are too long for extract, and we can only subjoin his conclusions upon this point. "1. With the exception of some whose position renders them inaccessible, it is almost always possible to remove these tumours. 2. In almost all cases such ablation is indicated, either for the removal of a great deformity, the relief of continual suffering, or the re-establishment of the deranged functions of certain organs. 3. In the great majority of cases we can proceed to the ablation without a preliminary exposure of the tumour, and without having any great difficulties to overcome. 4. Such an operation may lead to unfortunate results, in consequence of some peculiarity in the seat or relations of the tumour, or from the bad constitution of the patient; but generally it is crowned with success."—*Revue Medico-Chirurgicale,* Nos. 2 and 3.

[The above extract from the pages of our new contemporary and namesake is taken from a paper furnished by M. Roux from among the materials he is now engaged in arranging for his long-expected work on "Clinical Surgery." His well-known candour in stating the results of his experience, whether favourable or unfavourable; and the long period during which he has been accumulating facts furnished him by an extensive private and public practice, naturally give rise to the highest expectations.—*Rev.*]

---

### On Iodide of Potassium in Syphilis. By Dr. Aran.
(Archives, Vol. 13, pp. 77-161.)

In this paper, Dr. Aran enters into a critical examination of the most remarkable writings which have recently appeared upon this subject, as those of Dr. Moij-Sisovics, of Vienna, Dr. Hassing of Copenhagen, Drs. Gauthier, Payan and Bassereau. (*Med. Chir. Rev.* No. IX. p. 267). His appreciation of these seems to us to have been very conscientiously and carefully made, and we really hope that it will be for some time to come considered as final, for important as the subject is, it seems to us to have been at least sufficiently insisted upon of late. The following are Dr. Aran's conclusions.

"1. Frequently repeated experience has indubitably established the fact, that iodine and its preparations taken internally possess valuable anti-syphilitic properties. 2. The iodide of potassium is far preferable to the other preparations, and deserves a preference to them in almost every case. It is easily given, does not nauseate, frequently increases the appetite, accelerates nutrition, and is endowed with valuable curative properties. 3. It is not indiscriminately applicable to all the periods of syphilis, or the different symptoms which characterize it. As a general rule, we may say it especially succeeds against what Wallace has termed the *pustular or deep-seated form of chancre;* it is of remarkable efficacy, and nearly always heroic, against tertiary symptoms and those of the secondary ones which are tending to tertiary. 4. It is precisely in those cases in which mercury shows itself powerless, or of only feeble efficacy, or when the symptoms are of very long standing, that the substitution of the iodide for mercury best succeeds, and that especially when these symptoms occur in a more or less shattered constitution. In this way the iodide fills up a lacuna which was heretofore painfully evident in the therapeutics of syphilitic diseases. 5. The utility of the union of the mercurial and iodic treatment is at present an undecided question. At all events this mixed medication seems to be without inconveniences. 6. The iodide is equally efficacious in whatever vehicle it be administered. Experience has shown that, from 10 to 15 grains *per diem* suffices at the commencement, and that this may be gradually increased to from two to four scruples. 7. The occasional inconveniences which this medicine gives rise to (such as cutaneous irritation, and inflammation of the conjunctival, nasal, or buccal mucous membranes), must not prevent our employing it, especially as they may be in part prevented, or moderated, by careful administration."

We may here add the account M. Aran furnishes of the treatment pursued for several years by M. *Moij-Sisovics* senior physician to the Vienna Imperial Hospital, and described by him in his work upon "A rapid and certain Mode of treating Syphilis by preparations of Iodine;" and which, if the author's statements can be relied upon, certainly surpasses all others in prompt efficacy. "Half-a-drachm of iodide of potassium, dissolved in water and divided into three doses, is given the patient daily, the quantity being gradually increased until four scruples are reached, beyond which it is never extended. Baths of iodine and marine salt are simultaneously employed (salt 2 lbs., iodide of potassium 6 scruples, pure iodine, added when the patient is in the bath, 4 scruples) for an hour at a time, the patient lying in a hot bed afterwards until transpiration is excited. These baths are continued for three days until some irritation of the skin is induced, the quantity of iodine being then augmented. On the 10th or 11th day a febrile state comes on, itching of the skin is felt and a scarlatinoid eruption, or one resembling zoster, is produced. On the 15th, 17th, or 21st day a general desquamation occurs, which, with the previous eruption, indicates the saturation of iodine has reached it maximum, and the author declares he has never seen a relapse in a case in which the eruption and desquamation have pursued the regular course. For the exostoses, condylomata, and pustules, Dr. M. employs a strong local application formed of iodine, the iodide and water. He orders alimentary substances of easy digestion, taking care to avoid those of an amylaceous nature, and keeps the patients warmly clad in a room having a mild temperature. This treatment he employs against every description of syphilitic disease, primary or secondary; and he declares it is followed by a cure in from fifteen to twenty-two days."

---

### On the Use of Assafœtida, as a Means of preventing the Death of the Fœtus in Utero, during the Pregnancy of Delicate Women. By Dr. Laferla of Malta.

The author, from an early period of his professional career, has directed his attention to those cases in which the fœtus having reached its period of development dies prior to birth, the mother in this way sometimes bringing forth a succession of dead infants. In reflecting upon the subject, he felt disposed to attribute the occurrence to debility or inertia of the uterus, and in searching for means to invigorate the condition of this organ without inducing its contractions, he remembered the commendations bestowed by Sydenham upon *assafœtida* in hysteria, and especially in cases of debility of the womb. His employment of this in several cases has led to excellent results, which it is the object of the present paper to detail.

The fœtus may die at three different periods of pregnancy—before the sixth month, during the two first months of the third period, and when arrived at full term, independently of the nature of the labour. When a pregnancy is so menaced, there are several symptoms which should excite attention. At first there is a feeling of languor, general uneasiness, restlessness, horripilation, and a sense of great cold. The countenance becomes pale, the head heavy and confused, and the tongue loaded, vomiting or nausea even sometimes occurring. The spirits are low and the nervous system very excitable. The movements of the fœtus are languid, and at last only its cardiac pulsation can be perceived. There is numbness of one or both legs, and severe pains of the loins, belly, and pubis come on. The child feels a heavy weight in the pelvis, and a few days prior to its expulsion, a whitish-yellow or sanguinolent discharge takes place from the vagina. The *causes* of this state of things are usually depression, passions or frights, venereal diseases, a lax constitution, amenorrhœa, leucorrhœa, menorrhagia, hysteria, or a neglected miscarriage.

In apportioning the medicine the temperament of the woman and the period of her pregnancy must be taken into account. "To those of sanguineous or bilious temperament, from two to four grains per diem less should be given than to others; and I always take care that a pregnant woman, before arriving at the period at which she has already lost a child, shall have taken from 10 to 15 scruples of the medicine in all. At first I employed the tincture, but finding the pills more easily taken, I have since combined the powder of assafœtida with extract of chamomile, so as to make 2-grain pills. Of these I give one fasting, and another in the evening, five hours after dinner. This dose may be gradually increased as we approach the critical epoch; so that, if in prior pregnancies the child died during the first period, this should be done every second day; if on the second period, every four or five days; and if at term, every six or eight days. We have best chance of succeeding when we commence the treatment even before the pregnancy has begun. I then prescribe from 6 to 8 grains per diem, divided into two doses, until certain signs of pregnancy manifest themselves. After these are present, I then give but the simple 2-gr. pill night and morning, and if the movements of the fœtus are of normal strength, and the pregnancy does not offer the same deviations as the former, I continue the same dose to within about a month of the period at which death usually takes place. The continuous use of assafœtida gives rise sometimes to a burning sensation in the stomach. In this case I suspend it for some days, substituting some decoction of gentian until the heat is dissipated. Great attention to moral agents is requisite, and the strength must be well maintained by a succulent diet."

Dr. Laferla has met with about twenty cases in which he has tried this medication with success, and seven of them are here detailed. In some of these, three, four, five, and even eight unfortunate pregnancies occurred prior to the successful use of this drug.—*Revue Medico-Chirurgicale,* No. 3, pp. 129-137.

[M. Laferla has directed attention to a very important point and one too much neglected. We cannot agree with him in his opinion that a special corroborative power is exerted by the assafœtida upon the uterus, but believe that it may frequently be useful in counteracting that nervous susceptibility which women prone to this accident so commonly manifest. In another and a large class of cases the loss of successive children is referable to a syphilitic taint existing in either parent, which is removable by a mercurial course.—*Rev.*]

---

### Clinical Observations upon the Treatment of Squamous Diseases of the Skin at the Hôpital St. Louis. By M. Devergie.

*Arsenic.*—Although the various squamous affections of the skin present sufficiently distinct characters to require that they should be nosologically distinguished from each other, yet, in a therapeutical point of view, they may be considered in a more general manner. Arsenic may be given in the form of *pills* or of *solution.* The pills, known under the name of *Asiatic,* are thus composed: *Arsenious Acid* 1 gr., *Black Pepper* 12 gr., *Gum* 2 gr., *Water* q. s. to form 12 pills. One pill, or at most two pills, should be given daily for 6 or 8 weeks; but this mode of administration is so uncertain and so frequently induces irritation of the digestive organs that M. Devergie now never has recourse to it. Among the *Solutions,* that of *Fowler* (*Liq. Potassæ Arsenitis, Phar. Lond.*), furnishing a very soluble arsenite of potash, is best known. When I first came to St. Louis it was customary to give only one drop of this every 24 hours for a certain number of days, increasing this at very long intervals to 2, 3, 4 and even to 12 or 16 per diem. I found, however, that by giving 1 drop the first day, 2 the second, and thus increasing by a drop daily until the maximum (12 to 16) was attained, the duration of the treatment might be much abridged without any injury to the health. We have accounts of as much as 40 drops of this substance being given, but this must be erroneous, and a careful investigation has convinced me that we cannot give more than 20 or 22 per diem without producing injurious effects, and that 14 form the ordinary limit. This substance exerts its action both on the skin and upon the general economy.

*Action on the Skin.*—After a certain time the scales fall off, and although the ointments which are used detach these, it is the arsenic which prevents their reproduction. The skin gradually assumes its proper level, and the affected parts, from being red, become brown and smooth. This is of great importance, for it is only when we have obtained this *brown colour of the skin* we can be assured of the cure. It is the proof of the efficacy of the medicine and the measure of the quantity required. So certainly is this brown colour of the spots a sign of cure, that, if there is a relapse of the disease from a recurrence of its causes at the end of a year, or even of three months, it never re-appears upon the portions of the skin originally affected, but always on one side of them. The disease is cured when these spots are seen: but they require some months or even a year to disappear.

*Action upon the Economy.*—The first effect of arsenic is a general falling away of the whole body, and a brown leaden coloration of the face; from which it results that patients who have completed the course of treatment and feel themselves very well in all respects, have just the appearance of convalescents from serious disease. With no pain or other disorder of the economy, and possessed of a good appetite, they continue so thin as still to seem very ill. Arsenic would seem to *exert a modifying power upon the formation and secretion of fat:* and on this account I have prescribed it for the resolution of adipose tumours, and several times with success.

Arsenic sometimes gives rise to *certain accidents* which it is important to bear in mind. All persons cannot bear the same dose. In some, eight drops will produce anorexia and other disorders of the system, and in such cases we must suspend its use for some days, and only resume it very cautiously. It sometimes happens that when we have again reached six drops all the ill-effects are reproduced, and in this case we must definitively renounce it, for not only may it do much mischief but it will not cure the disease against which it is employed. There are other subjects in whom 12 drops produce accidents which show that the drug has acted sufficiently, and must be left off. These are symptoms which have not been hitherto indicated by authors. Some patients will tell you they suffer from dyspnœa, others from partial loss of strength, as in a limb, others from colic or diarrhœa, from numbness of a limb—in a word, from one of a varied series of more or less strange nervous phenomena—none of these being symptoms characteristic of poisoning by arsenic. Of all these dyspnœa is the most constant. Symptoms of true poisoning may, however, occur, these arising when too large a dose has been given. The presence of this medicine in the urine would prove that the system is saturated with it, and that its therapeutical action had reached its limits.

The arsenic is best given in two doses per diem in a julep or sugared water. M. Devergie employs a far weaker solution of arsenic than Fowler's, so that 22 drops of it represent 1 drop of the latter. He does this because the quantity can thus be more easily measured out; and accidents are less liable to occur from a mistake in the number of drops. *Pearson's Solution,* which contains an arsenite of soda, is less easily supported by patients than the above.

*Antimonials.*—These may be also administered in two manners. In a pill, (*Plummer's Pill),* each pill containing one grain of calomel and of the sulphuret. Of these we may give from two to six daily; but, as with respect to arsenic, the pilular form is a bad and uncertain one, on account of the insolubility of the materials, I therefore much prefer *tartar emetic.* I mix half-a-grain of this with from half-a-drachm to a drachm of *cream of tartar,* and give them mixed up in preserves, the patient swallowing a glass of sugar and water directly after. It is remarkable that, given in this way, these medicines produce no vomiting, and scarcely any purging. The toleration is indeed surprising, and it is as much as there is a little nausea the first few days. In those cases where vomiting or purging is caused it must be left off. This treatment requires to be continued in general for two months, and is not evacuant but alterative in its operation.

As M. Devergie considers *external treatment* of these diseases as of comparatively little use, we need not detail the various means at any length. Of these he seems to think the application of *tar* (melted down with at first 40, and then

20, 30, 10, or 5 parts of lard), night and morning, as one of the best, when used in combination with baths, the patient always sleeping in the same shirt and bed-clothes. The ointment has to be employed for several weeks, and if it is to prove efficacious, a white line will be seen after a while to form around the circumference of the spots, produced by the decoloration of this. In the varieties of psoriasis termed *guttata, gyrata, orbicularis,* &c., it is from the centre to the circumference that the whiteness and the cure proceed. *Alkaline* ointments are best formed of *carb. sodæ* (from 2 to 8 parts previously dissolved in water to 30 of lard), and will sometimes cure a psoriasis which has resisted tar. Indeed *lard* will occasionally do this, and should be generally employed for children either alone or feebly medicated. *White precipitate* (2 to 6 parts to 30 lard), is not unfrequently successful, but it may, if the surface is an extensive one, produce salivation, a result less likely to follow the use of *calomel ointment* mixed up with a few grains of camphor. *Vapour* or *alkaline baths* should be conjointly employed with the above means, the latter being formed with carbonate of soda, to which salt is added, if a tonic, or gelatine if an emollient effect be desired.

In choosing among the various modes detailed several circumstances have to be considered. 1. If the disease is *hereditary,* whatever treatment we adopt we shall rarely produce a complete result. 2. If the patient possesses a strong constitution, we must not fear employing perturbatory medicines, which would be improper for weakly habits. 3. We must learn the duration of the disease, and whether it is a relapse. If it has never yet been treated, we must use every endeavour to procure a radical cure; but if it has been already treated we must fear its re-appearance, and, for its temporary dissipation in this case the external treatment is especially indicated. 4. Whenever the lepra or psoriasis is of recent origin, especially if occurring in a robust patient, from the internal treatment (arsenical or antimonial) we may expect a radical cure, providing always that the patient afterwards takes as much care as possible of the skin. If the patient is weakly, sickly, or of a bad constitution, we must be content with the external treatment.

In young, delicate, and feeble subjects, the *hydrotherapæic method* has a corroborant effect. By its aid I have cured cases of very long standing. The patients in general, too, bear it well, and under its use, in spite of the sweating induced, they gain flesh, probably in consequence of the more vigorous appetite that is produced. In most of the cases I have so treated, however, relapses have occurred in the course of a few months; so that it seems to possess no more power of *radically* curing the disease than the other external measures.—*Gazette des Hôpitaux,* 1847, Nos. 1, 13, and 22.

---

### Observations upon Itch. By M. Devergie.

M. Devergie believes that some of the generally-received opinions concerning this disease possess a very insufficient foundation, and, after long and attentive observation in the wide field at his disposal, he has arrived at the following conclusions.

"1. There is a very general opinion prevailing in the world, that the itch is the cause of the skin diseases which may subsequently occur. Without attaching too much importance to this, does it not rest on more or less probable foundations? 2. Although itch is said to be essentially contagious, it may yet frequently arise spontaneously in individuals placed in similar conditions under which it was primarily developed. 3. There is no proof that it has been transmitted from animals to man. 4. When medicines cure the itch, it does not follow that this is by destroying the acarus. The cure of the pustules may be rather said to lead to its death. 5. This insect may just as well be considered as a morbid product as a cause. 6. The experiments undertaken to show that the contagion is operated by means of the insect, offer insufficient proof of this being the sole means of infection. 7. In the hypothesis of contagion being due solely to the acarus, as the contact of individuals with each other does not always take place in the same manner, we must suppose that the acarus for a certain period crawls over a large portion of the surface of the body to the place of election (the hands and feet), to the neglect of many other portions, which a few days after will be covered with itch-pimples. This regular and simultaneous development is much more likely to be dependent upon a general cause acting upon the entire economy, than upon a local one, arising from an insect transplanted from one individual to another. 8. If the acarus is the cause of the itch-pimple, it seems difficult to understand how it escapes from the central point of the pustule while digging a deeper gallery beyond it, scarcely ever having any communication with another pustule. This fact, which may be daily observed by the naked eye, is much more in harmony with the hypothesis of a morbid generation. 10. There is nothing fixed in the incubation of itch, which is much more in accordance with what takes place in other cutaneous diseases than with the idea of infection by means of an insect. 11. It is in the severest form of itch, the pustular, that we find the fewest acari. This form is cured most easily, and seems the least contagious. 12. It is singular to find the same insect producing three different forms of eruption; and not only are these forms different, but so are their contagious properties and the number of insects found in them. 13. Statistics show us that itch is the most common antecedent of impetigo, lichen, and eczema. 14. The itch may disappear for a greater or shorter period under the influence of a general affection of the economy, and, like the other cutaneous diseases, remain latent, and then re-appear with renewed energy after five or six weeks, without any new infection having taken place."

From these corollaries M. Devergie draws the following conclusions :—

" 1. If the acarus is one of the phenomena of itch, its existence as a morbid product is as admissible as an agent of transmission. The known facts agree much better with the former hypothesis than with that which considers the insect as the exclusive agent of transmission and only cause of contagion. If even the acarus, by being transported to another individual, may communicate the disease, the products of secretion, the itchy atmosphere, and the clothes impregnated with this, may also produce it. 2. The principal therapeutical consequence deducible is, that we must treat the itch, and not the acarus, contrary to what is generally the practice. We should treat the disease like other cutaneous affections upon general principles, and not by mere local applications. Indeed, is it not reasonable to take into consideration the two well-marked symptoms, whose sudden suppression is so mischievous in other cutaneous diseases, viz. the *itching*, become habitual in proportion to the duration of the disease, and the *secretion* in the pustular form. In the place of searching for means to cure the itch in the shortest possible time, should we not endeavour to do so gradually in proportion to its duration? Should we not, after its cure too, prescribe in some subjects the prolonged use of simple baths, to reduce the morbid sensibility of the skin and re-establish its functions, or even vapour-baths to produce a sedative effect on the nervous system, as well as a depurative cutaneous secretion, if I may be allowed such an expression? So, in regard to pustular itch, should we not act revulsively upon the intestinal canal by means of purgatives, to compensate for the suppression of secretion over so multiple a surface? This is my practice, and I do not feel disposed to abandon it, because it seems a rational means for the prevention of the ulterior development of other cutaneous diseases, of which I believe itch to be one of the predisposing causes."—*Gazette des Hôpitaux*, No. 32.

[M. Devergie seems to us, in the above observations, to have raised a very important point for consideration. That the doctrine of the exclusive propagation of the itch by the acarus is baseless, we have long been convinced; but we must confess that we have not hitherto viewed the rapid suppression of the disease in any other light than a very desirable circumstance, seeing the great amount of bodily and mental irritation its persistence keeps up. The employment of baths and purgatives, however, after the cure, as recommended by M. Devergie, is a most rational procedure, and one far too much neglected.—*Rev.*]

---

### A Sketch of the Life and Works of James Tommasini,* read before the Royal Institute of Venice. By Professor Giacomini.

It is very remarkable, I may say almost marvellous, that among so many justly celebrated men in Italy, so many *savants* desirous of renown; amidst such a diversity of principles as prevails in our peninsula, it is agreed on all sides, without even excepting his adversaries, that Tommasini may be considered as the foremost, the most experienced, and the most profound clinical observer of our epoch in Italy. The true reason of this general fact is found in the immense services which this great man has been rendering for more than half a century to his art and to society, whether as a professor, a writer, or a practitioner. To him in fact we owe the happy direction which medicine has received throughout Italy from the commencement of this century, the greater part of our best clinical observers having been formed in his school. Elected at an early age to the professorship of physiology and pathology in the University of Parma, he exhibited great intellectual power and practical tact in the first work he published (*Critical Lectures in Physiology and Pathology*, 1802). Even now we admire in this work deep sagacity, healthy criticism, practical deductions of great importance, and an erudition alike rich and in good taste.

A short time after a wider field of observation presented itself to the investigating spirit of Tommasini. We allude to his clinical labours. This epoch was full of events of the highest import to Italy. Natural philosophy, restored by the labours of Bacon and Galileo, had produced so much embarrassment among the prevailing medical doctrines, that physicians gifted with more than ordinary intelligence, acquired a melancholy scepticism respecting practice. And thus no sooner had Brown's seductive doctrine appeared above the horizon than it was welcomed with avidity, and in a short time medicine in every country was found to be modelled on the views of the Scotch philosopher. Rasori was the first who made known the principles of the new school in Italy, by translating Brown's "*Compendium*:" and he it was who first, towards the end of last century, clinically demonstrated its errors. The Scotch physician had laid it down as a principle that all agents applied to the living tissues exerted upon them a stimulant effect, and that diseases emanated from defective vitality, as was made apparent from the loss of strength which was observed in all diseases. Rasori exposed the errors of this doctrine by proving, as Hippocrates had already established, that the greater number of diseases which lead to death induce in the organism a condition of excess of the vital powers; and demonstrated by "solemn" experiments at Genoa and Milan the law of morbid capacity and the existence of medicinal substances which act positively and directly by depressing the organic forces, and modifying the organism in a manner directly opposed to that of the action of stimuli, and which for this reason he has termed *contra-stimulants*. From these very simple principles, based upon experience, the medical

---

* Tommasini was born in Parma in 1765, and died there in 1846.

reform operated by the powerful genius of Rasori in Italy, took its origin. This great observer, however, carried away, after the discovery of these fundamental facts, by the whirlwind of political events, was compelled, for a long time, to leave his official post at the hospitals, and live in a kind of obscurity, after having gone through the severest trials during the course of those revolutionary times: so that the primary basis of the medical reform might have remained unimproved, and without a worthy interpreter, if Tommasini, who was the fellow-citizen and fellow-student and rival of Rasori, had not taken possession of them, to develop and confirm them by new experiments, and place them in the full light of day, with all the importance they deserved. Tommasini was in fact the first who exhibited in relief the immense practical consequences which flowed from the new facts discovered by Rasori, and he occupied himself by means of numerous observations upon a great number of medicinal substances and diseases, in proving their reality. He has in this way really enriched Italian medicine with a great number of novel facts, which have thrown the greatest light upon its practice, and led our physicians upon a way of investigation entirely different to that which they had hitherto followed; and this assemblage of facts has constituted so compact and immoveable a phalanx, that he has been able, since its accumulation, to defy his adversaries with impunity, and to resist, with a brilliant success, all the attacks which the advocates of retrograde opinions have incessantly directed against him.

Consequently it is to Tommasini, beyond all others, that we owe the new reform in Italian medicine—a reform which has continued progressive from its commencement, and which is incessantly extending itself in the medicine of all countries in which practitioners take for their guide true experience and the precepts of natural philosophy. The entire proof of the truth of what I advance is to be found in the numerous works themselves of our illustrious countryman. It will suffice if I refer to those most directly bearing upon my subject; and it will be easy for me to prove, that the new facts with which he has enriched science, have influenced the practice of our art among all civilized nations, not excluding even that of his own adversaries, who have adopted them, masked under a different phraseology. We may first refer to his experiments and observations upon various remedies, especially digitalis, commenced in 1804. Any one reading these, and the long and varied discussions they gave rise to on the part of his celebrated adversaries, will find wherewith to become edified upon the subject of experimenting and the true logical method of drawing deductions from experiment. He will see that the experience which every one invokes with a sort of ostentation, I may say to a tiresome extent, in support of his own opinions, is itself, in the absence of philosophy and criticism, the first source of error. At the present day, when time has pronounced its verdict upon the famous question which cost Tommasini and his adversaries so much pains and labour, viz., *whether digitalis exerts upon the economy a stimulant or sedative effect*, we feel astonished that so evident a fact as the contra-stimulant power of this drug should have had so much difficulty in making its way. And yet the fact in relation to the action of digitalis, which is now generally recognized, is no less evident in respect to a crowd of other contra-stimulant remedies, concerning which observations, just as numerous and conclusive exist—observations which the obstinacy of some adversaries persists in neglecting, substituting with a desperate constancy preconceived opinions to the material facts which alone should be invoked in questions of this nature.

In the middle of the polemics which overturned Brownism in Italy, Tommasini published his monumental work, *Pathological Researches on the Fever of Livorno, the Yellow Fever, and other analogous Diseases*, 1805. In this were discussed and resolved the most important questions in relation to fever, and the true pathology of gastric and bilious fevers, and the doctrine of the diffusion of local inflammations established. He therein, too, signalized the true material condition of continuous fever, and liberated for the first time the numerous family of fevers from the empire of the abstraction and the essential nature. He demonstrated that the phlogistic process was always the same in its forms and consequences, in its acute as in its chronic progress, and he laid down general precepts, which have become inviolable practical laws in Italy, for the exact appreciation of diseases in clinical practice—precepts which recal to mind those contained in the works of Hippocrates, of which Boerhaave said *Quo plus evolvo eo ditior in praxi fio!*

The various works published subsequently by Tommasini are but amplifications, based upon a much more extensive practical experience, of the principles expounded in his "Researches"; so that we must refer to the epoch of that work, and of that published upon the Genoa epidemic in 1799 by Rasori, the commencement of the great reform in Italian medicine—a reform really based upon Hippocratic principles, or in conformity with these principles and with those of experimental philosophy which the doctrines of Brown had caused to be completely forgotten in Europe. This work of Tommasini's went through a great number of editions in a short time, and was translated into several languages; and I must here advert to a fact of importance as regards the history of the medicine of our times. While this work was in the hands of all our practitioners and justly appreciated by them, the celebrated Broussais was then with the French armies in Italy, and sojourned for a long period with us. Returning to France, this illustrious physician published, in 1808, his first work on the *History of the Chronic Phlegmasiæ*. This work is but the echo and copy of the doctrines of Tommasini concerning chronic inflammations and the nature of gastric fever and of fever in general; with this sole difference, that while the great Italian physician placed the seat of fever, according to the symptoms, in a phlogosis of this or that viscus, and frequently even in a diffuse inflammation of the entire sanguineous system, the celebrated Frenchman located it exclusively in the gastro-intestinal canal. Broussais could not be ignorant of the opinions of Tommasini upon this subject, nor have omitted the careful study of his works; and the just reclamations which were raised in Italy upon the appearance of his work, obliged him to offer an explanation in his "Annales de Medicine Physiologique." He acknowledged his acquaintance with the works of Tommasini while in Italy, and admitted that he had been preceded by him both in his researches and his conclusions. In the mean time French pathology (not so therapeutics, for unfortunately Broussais had not availed himself of the studies of the Italian school upon medicinal substances, but retained all the old aberrations of Brown respecting these) received from the labours of Broussais a vigorous impulse. This physician created for himself an European reputation, and his sarcastic language in the end obliged his very adversaries to change their principles. And strange to say, Broussais, who had only borrowed his pathological principles from Tommasini, acquired partisans in Italy among the very persons who had rejected the conclusions of Tommasini!

In all the various works of Tommasini, whose titles even I cannot enumerate, there will be found pervading each page a judicious and practical spirit, founded upon the works of Hippocrates, full of sagacity and wisdom, enlightened by a prodigious erudition, and enlightened by that probity and love of truth so delightful to find in men of science. We can, therefore, in no-wise feel astonished that, as a practitioner, a writer, or a *savant*, he has always been considered, both in Italy and elsewhere, as the true patriarch of Italian medicine, just as he was really its regenerator. In his clinical histories published in so many of the Italian journals, he was most especially careful in the relation of unfortunate cases, accompanying them with considerations and warnings of great practical interest, and concluding with valuable remarks upon the causes which had led him or might lead into either error or doubt as to accurate diagnosis or treatment. It was in these cases that his enlightened circumspection and great practical sagacity were so conspicuous. I do not so term that impassable inertia, which, decorated with the name of prudence, allows dangerous diseases to proceed on to their fatal termination, and then washes its hands of them; so that the disease may bear all the blame and the practitioner be protected from all reproach; the vulgar being always disposed to blame as culpable the active intervention of the physician when this proves insufficient to save the patient's life! In these most serious and difficult cases, Tommasini employed an unheard of zeal in combating the disease with boldness and vigour until the very limits of art were reached, and he frequently succeeded in triumphing over it in spite of the desperate circumstances amidst which he was employed. Tommasini was always in the habit of preparing statistical tables of his clinical practice, and first taught the true manner of turning these to practical account, protesting against, as a vain and inconclusive practice, the simple accumulation of figures.—*Gazette des Hôpitaux*, No. 27.

[The fate of Brownism in Italy constitutes a curious feature in the medical history of our epoch. Rejected in this country and in France, it was welcomed in Germany, and especially in Italy, with the most flattering reception. Rasori constituted himself one of its earliest and most ardent interpreters; but his practical sagacity soon taught him that, however seductive from its simplicity the system of the Scotch theorist might prove; that so far from the vast majority of diseases being of an asthenic nature, proceeding from debility of tissue, and requiring stimulant remedies, the very contrary is far nearer the truth. Hence the re-action described by Professor Giacomoni in the above paper, which has since pervaded Italy, as it did France, under the *prestige* of Broussais. In both countries the other extreme was run into, and a far too indiscriminate blood-letting was the consequence: but while in France a more rational state of things now prevails, in Italy the antiphlogistic school seems still to exert undisputed sway. The charge of plagiarism brought against Broussais will be new to some of our readers, although it seems well-founded.—*Rev.*]

---

### Practical Observations upon Foreign Bodies introduced into the Eye. By M. Petrequin, of Lyons.

M. Petrequin divides these bodies into three categories, those which are arrested on the surface of the eye, those which become imbedded in the cornea, and those which, perforating the cornea, penetrate more or less deeply into the chambers of the eye. In a manufacturing town like Lyons these accidents are very common.

1. *Foreign Bodies arrested npon the Surface of the Eye.*—These often become hidden under the upper eyelid, and are of difficult removal by the usual plan of raising the lid upon a probe, &c. M. Petrequin employs a camel's hair pencil, and introducing it beneath the eye-lid, he passes it from the one commissure to to the other, so as to sweep any foreign body into the nearest angle of the eye. If he has not a pencil at hand he employs the feather of a pen. It acts best when used dry, the tears sufficing to moisten it. Extraction in children is difficult in consequence of the convulsive contraction of the eyelid, which often occurs, so that even opening these at all may be impossible. M. Petrequin throws in an injection of rose-water to dislodge the body from the corner of the eye where he supposes it placed, and brings it thus easily to the free edge of the eyelid. Practitioners should always recollect that the sensations induced by a foreign body may continue after it has been removed, so that they may not needlessly make painful researches.

2. Workers in iron or stone may have particles fix themselves to a greater or less depth in the substance of the cornea, and these are sometimes so small as to require a lens to see them in the little depressions in which they are seated. If the particle is metallic, we should try the effect of a magnet for their removal. M. Petrequin considers a forceps a bad instrument, for, however delicate it may be, we may try 20 or 30 times without being able to seize the body with it, irritating the eye more and more, and increasing the difficulty of the extraction. The case is rare for the body to be large enough to have a free portion sufficient for seizure. The bistoury is preferable, but it should be a rounded one, for with a sharp-pointed one, we might risk piercing the eye during one of its spasmodic movements. M. Petrequin prefers a large lancet, the front and edge not being too sharp, and its form resembling that of an abscess lancet. The patient should be seated in a chair, the arms of which he grasps. The light coming from above, he is directed to throw back his head until its long diameter becomes very oblique from above downwards, and from behind forwards. The surgeon placed behind supports it against his chest, and directs the patient to look upwards. After having raised the upper eyelid he directs the cutting edge of the lancet very gently and carefully upon the foreign body and slightly scrapes the cornea. The eye immediately becomes convulsed and is retracted beneath the lid. It is brought down again by looking at a fixed point, and the scraping again commenced. In this way the eye becomes accustomed to the contact of the instrument, and the foreign body is easily removed. It must always be shown to the patient, since, on account of the continuance of the pain for some hours after, he may doubt the fact of its extraction. After the operation, a plastic effusion takes place into the little cavity which becomes filled up by its organization, no trace of cicatrix remaining if the operation has been adroitly performed.

3. These cases are always grave, as the contusion produced may give rise to traumatic amaurosis, and at all events to inflammation. Two or three cases of this accident are related, one of which we abridge. A countryman, æt. 50, was admitted into the Hôtel Dieu on account of a foreign body which had entered his eye while cutting stone. This occurred some time before his admission, although how long is not stated, and the patient had been submitted to means for the relief of the attendant inflammation with little success. The cornea was entirely healed and offered no trace of cicatrix, and through it the bit of stone, not larger than a millet seed, was observed to be lodged in the anterior chamber between the cornea and iris, at its superior external part. The conjunctiva was much injected, and the iris inflamed. It acted imperfectly, and some adhesions to the lens had occurred. Vision was defective, and attended with great pain in daylight. M. Petrequin made an incision four lines in length, into the upper and external fourth of the cornea. The aqueous humour flowed out, and the iris presented itself. He passed in a pair of Sichel's cataract forceps closed, and opening one blade above and the other below the body, he seized it with some difficulty on account of the little support the iris furnished it, this receding from the instrument. A small portion of the iris which was prolapsed was removed by the scissors. The patient was bled after the extraction, and he went on quite well, perfect vision having been recovered. A small hernia of the iris was removed by cauterization with the nitrate of silver.—*Annales d'Oculistique,* T. 17, pp. 14-20.

---

### On the Dentition of Children, from the Clinical Lectures of M. Trousseau.

The most recent works upon Odontology contain some errors respecting this process, which it is the duty of the clinical teacher to indicate. The *evolution of the milk teeth* is not usually accurately described as respects the order of sequence. M. Trousseau represents it as occurring in groups, separated by pauses. 1. The first group is formed of the two *lower median incisors* which appear nearly simultaneously, after which there is a pause for one or two months. 2. The two *upper median incisors* pierce the gum from the 10th to the 12th month. 3. The third group follows the second in a fortnight or a month, so as often to be confounded with it, and consists of the two *upper lateral incisors* (not the lower ones as usually stated.) 4. Again is there a pause, and we then find the *lower lateral incisors*, and the *four first molars* appearing almost simultaneously: first a molar generally shewing itself, then an incisor, a molar again, and then the other incisor, and lastly the two molars. 5. After a pause of from two to five months the four *canines* appear; the 6th group, consisting of the *second molars*, scarcely ever appearing until five or six months still later. This is not a matter of mere curiosity; for the occurrence of these pauses should be borne in mind, *e. g.* in weaning. For this we should choose the intervals between them, and above all avoid attempting it just at the period of the evolution of the 4th group. The two first teeth generally appear from the 7th to the 9th month; the upper median incisors from the 10th to the 11th: the upper lateral ones at the end of the 1st year. About the 16th or 18th month the child should have 12 teeth; 16 from the 20th to the 24th month, and upon the appearance of the second molars, generally about the middle of the third year, twenty.

In the course of the 6th year, and even sometimes not till the commencement of the 7th, but rarely before the 6th, we see a large quadricuspid persistent molar appear: so that at seven years the child usually has 24 teeth; and it is at this time the milk teeth begin to be shed, generally in the order of their evolution. Thus the lower median incisors fall at 7 years; the upper ones at 8; the first molars in the course of the 10th year; the second molars towards the 11th; and the canine between the 11th and 12th—all these teeth being replaced by the corresponding persistent ones. At 12 we see the second large molars appear, and the child has then 28 teeth: and towards the age of 20, 25, or even later, the wisdom teeth appear, and the number found in man (32) becomes complete.

It is commonly said that the first persistent molars appear at the fourth year; but this is an assertion contradicted by daily observation; as neither their eruption or the shedding of the milk teeth scarcely ever commences prior to the 6th year.

The teeth whose eruption is least frequently attended by derangements of any kind are, beyond all others, the incisors, which is generally attributed to their cutting form; and the most difficult of all to cut, notwithstanding their conical form, are the canine. They are deeply seated, and, as it were, wedged in between the lateral incisors and first small molars, which they are obliged to push on one side. The eruption of the molars is rarely attended with much inconvenience. We should always discourage weaning during the period of the eruption of the canines.

Four or five days prior to the eruption of a tooth there is usually a slight catarrh, cough, some dysphagia. Frequently its cutting is accompanied by diarrhœa, loss of appetite, and great pain. The gum is usually inflamed over the tooth; and a little later this inflammation is also seen around the teeth already cut. Some children also do not cut any teeth without paroxysms of convulsion; and in some cases these form the first indications of what is taking place. It is a general practice to attempt relieving the inflammation of the gums by lancing them: but in 24 hours the incision heals, and the subsequent cutting of the teeth becomes more difficult and painful in consequence of the cicatrization. The tooth in fact, often appears far nearer the surface than it really is, and very erroneous views prevail respecting its mode of arriving there. They come forward so slowly that they have time to modify the tissues. They do not distend the gingival membrane, but inflame it. Frequently at the summit of a tooth which is not yet cut, we may see a little projection, which disappears after a while. It does not depend upon the pressure of the tooth, but upon an inflammation which renders the gingival tissue more permeable, and its absorption more easy. If this slight tumefaction was the result of pressure, the tooth would make considerable progress in a short time; while, after the gum has subsided, it is hardly apparent. If convulsions are present, we should employ immersion in cold water, antispasmodics, narcotics, or laxatives, according to circumstances. Diarrhœa is a more serious symptom, acquiring great obstinacy during dentition; and is especially to be feared if weaning has been performed just at the period of the eruption of one of the groups.

In young children the teeth become carious more easily than in adults, and the caries sometimes gives rise to most severe pain, and to the detachment of the dental follicles, rendering the caries of the persistent teeth also more easy of occurrence. Should we extract such teeth? If we extract milk teeth in a child of two years old, the persistent ones appear sooner, but the alveoli contracting, the arch of the jaw becomes diminished in extent. The persistent teeth have then little room, and lap over each other. If we want the second set to appear regularly, therefore, we must not draw those of the first—at least only in cases in which the caries gives rise to very serious symptoms.—*L'Union Medicale*, No. 33.

[Although accustomed to attach much weight to the opinions of so sensible an observer in the enjoyment of a large field of practice as M. Trousseau, we should feel very indisposed to abandon the practice of free lancing the gums during any of the disorders of dentitition.—*Rev.*]

---

### Albuminuria in Pregnant Women.

M. Devilliers recently communicated to the Society of Medicine of Paris the results of the investigations which M. Regnault and himself have been engaged in making concerning the Albuminuria of Pregnancy. In this condition the affection presents much less distinct and well-defined symptoms, most of which may be readily confounded with those of other diseases of pregnancy. Sometimes no morbid phenomenon manifests itself; and at others there may be heaviness, headache, general uneasiness, some characteristicless derangements of the digestive organs; but no febrile action belonging to this disease in particular. Lumbar pains are so common in pregnancy, that we are unable to distinguish them either by their seat or nature from those which are developed during the acute stage of albuminuria. As to the dropsy, sometimes it does not exist, which however is rarely the case; sometimes it is limited to the lower extremities, resembling the simple œdema of pregnant women; and sometimes it is general, and may be mistaken for anasarca dependent on affection of the heart, which, during gestation, is frequently increased to a great extent. There remains then the albuminous condition of the urine: and, notwithstanding what various writers have stated, these enquirers have never been enabled to detect albumen in the urine of the pregnant women who have been presented to their notice under ordinary circumstances. In examples of the disease the quantity of albumen has been found very variable, increasing sensibly during notable disturbance of the circulation, *e. g.* during the development of intercurrent febrile affections, the period of accouchment, puerperal diseases, or the approach of death.

The affection which especially presents itself as a frequent and important complication of albuminuria is eclampsia. Most of the subjects of puerperal convulsions exhibit evident marks of serous infiltration. In some, however, this symptom is absent; but the authors of this memoir have *constantly* found albumen in their urine. Of course all the women exhibiting albumen do not become the subjects of convulsions; and the authors have found these absent eight times in twenty cases; but they believe that more than a mere coincidence exists between the two diseases, founding their opinion upon the fact, that in ordinary albuminuria various cerebral and nervous phenomena are exhibited, and that during pregnancy these last acquire a special development.

Contrary to what is observed in ordinary cases, the albuminuria of pregnancy may terminate in a rapid and complete cure after confinement, however grave the case may have appeared. It may pass into the chronic state, but this is more rare. It predisposes to the same affection in future pregnancies, it may induce abortion, seems to favour the development of puerperal diseases, and at the very least must be considered as a powerful predisposing cause of eclampsia; but of itself, and alone, it does not seem capable of inducing a fatal termination. The prognosis for mother and child is therefore only grave in proportion to the amount of complications. The authors observed 11 deaths in 20 albuminuric women.

Besides the various cadaveric lesions derived from the complications, the authors almost always found renal lesions of various kinds, and not exclusively such as are described as characteristic of Bright's disease; and they feel much disposed to acord to these organic lesions much less importance than is ordinarily attributed to them, and to regard them rather as the effects than the causes of the disease. Indeed, in pregnant women its causes seem to be different to those generally admitted in ordinary albuminurea.

The researches of the authors have proved to them that the blood of women during gestation exhibits a remarkable diminution of albumen, especially in the latter months; a condition which Andral has shown to be favourable to the production of dropsies in general, and one which bears the relation of cause to effect in respect to the present disease. The albumen dissolved in the blood is, in the normal state, combined in certain proportions, with a certain number of saline materials, which allow the blood to traverse the canalicules of the breast, to be there submitted to depuration, without the transudation of a single trace of albumen; but if from any general cause, from a disturbed condition of the asssimilatory powers, the static equilibrium is destroyed, the renal parenchyma may become traversed by anormal elements, and albuminuria induced.—*Revue Medicale,* Vol. I., 1847, p. 449-52.

---

### Hygienic Rules to be observed in Myopia. By Dr. Sichel.

Congenital short-sightedness exists seldomer in an advanced degreee than in a condition of simple predisposition or slight commencement; and experience proves that it generally diminishes with the progress of age, if nothing be done to increase it, and if, so far from opposing the physiological actions of the organ, we favour their evolution. To this end the power of accommodation of the eye should be continually exercised, by removing the objects upon which it is habitually employed to as great a distance as possible. Especial care should be taken not to fix the sight for too long a time upon too small objects, or to employ it in reading too small letters, whether printed or written. These negative conditions are not sufficient. The sight must also be much exercised on large distant objects, the use of spectacles must be delayed as long as possible, or those of the weakest powers chosen, so as to give merely a greater clearness to objects without altering their size. They should also, even when the myopia is much advanced, be employed exclusively for seeing at a distance, laying them aside for reading, writing, or working. They are not required in the house or in any locality sufficiently known to pass along without difficulty. If the nature of the occupation or the degree of the myopia compels their use, their power should be much lower than that of those which are employed for seeing at a distance. Frequently we are obliged to allow concave glasses to young persons for the purpose of deciphering music or of following the demonstrations which are made upon a black ground in the schools and colleges; but it is very important that the number of the glasses should only be such as just to allow of vision at the desired distance, and their employment should be limited to this. Far better is it, indeed to do without them, and endeavour by frequent exercise to gradually increase the range of vision. We must not attach too much faith to the alleged impossibility of seeing without glasses; for abundant resolution and perseverance will frequently overcome every obstacle. If, during writing, the chest becomes fatigued by the bent position the state of vision obliges the young person to assume, a moveable desk may be employed, which may be raised or lowered as desired, and by increasing the visual distance a very slight determinate extent every week or fortnight, we can gradually increase the range of vision. The same contrivance may be employed for holding the music at the piano, and the injurious bending forwards thus prevented. Such means are applied with difficulty to large commercial books, and to the open books employed while learning the violin: and in these occupations, if the myopia is considerable, glasses are required. If the individuals are very young, we should postpone the period of their so employing themselves, and in the mean time exercise their eyes in such a manner as may tend to the diminution of the myopia. When this is impossible, let the glasses be as feeble as possible, and accustom the wearer at intervals to lay them aside and cast the eyes on distant objects, which will at the least have the effect of preventing the sight getting worse. These short-sighted persons must very seldom look through dioptric instruments, such as magnifying glasses, microscopes, &c.

"I may cite my own case. I was myopic from birth to a considerable degree; but I have, by my frequent exercise of the eyes, and by my care to allow myself the use of spectacles as seldom as possible, which in my youth exposed me to much privation and frequent ridicule, been enabled to render my sight twice as good as it was; for, in fact, I now read at a distance about as great again as I did twenty-five years ago."

The myops then should exercise his faculty of exercising the eye at different distances as much as possible, or he will lose the power of doing so, and find his affection gain upon him more and more. It is especially necessary to prevent young children approaching objects too near their eyes, and not to give them too small toys which will oblige them to do so. Not only will this cause an increased myopia, but it may produce strabismus. The visual axes naturally converge when we fix our eyes upon near objects; and the more this act is repeated the more habitual does the convergence become, until it ends in becoming permanent and passing into strabismus, It is on this account that this deviation of the visual axes is so easily produced between the ages of four and six, the period at which children are generally first taught their letters; and it especially occurs when there is an inequality in the focus of the two eyes.—*Annales d'Oculistique*, Tom. XVII., p. 200.

---

### Practical Remarks on Bleeding, and upon its Utility in the Treatment of the Diseases of the Uterus. By M. Tanchou.

*On Bleeding in General.*—It is not always easy to decide upon the institution or the repetition of bloodletting, and I have long been in the habit of consulting the condition of the blood itself for clearing up any doubts. Generally it is *black*, and that colour, in conjunction with its consistence, justifies its abstraction. Oftentimes, however, it is red at the commencement of the bleeding, and becomes black at the end of this. Again, it is at first black, and then becomes red; in a fourth case, the two threads of the spiral it forms may be of the two different colours, and lastly, the blood may be red from the beginning to the end of the bleeding.

Whence is this last due? There can be no doubt that when the individual is weak, and exhausted in some degree by preceding bleedings, it arises from the rapid and freer passage of arterial blood into the venous system; but when the patient is strong and young, the circulatory system full, and it is observed at the first bleeding, I believe it arises from the contact of the air, which, traversing the skin, acts upon the blood; for I have made this remark especially in hot weather, when the patients have been several hours in bed, upon young subjects affected with inflammatory fever, and young healthy pregnant women—whenever, in fact, the skin is supple, elastic, and perspirable, conditions favourable to the absorption of air; or in individuals suffering from exhaustion from any cause, and in whom the organism always consequently manifests an avidity for whatever may maintain life and recruit strength. I may remind you that the blood of infants is generally red, that of old persons black; and that the skin, under these two circumstances, is in quite opposite perspirable states.

However this may be, I have been led to the following practical inductions. If the blood is black during venesection we may allow it to flow, or repeat its abstraction if the symptoms indicate this; while, if it is red, we must absolutely abstain from doing so, or we shall only aggravate the symptoms for which we employ it, and increase the fever. Thus, in pneumonia the hreatbing becomes more laborious, and the oppression greater, as if the pulmonary congestion increased as we diminished the general resistance by the loss of blood. So in hæmoptysis the blood in the expectorations becomes redder and more abundant; and in metrorrhagia the flow is increased. In typhoid fever bleeding is of use when the blood is black, and constantly hurtful when it is red. The remark should not be lost sight of by those who practise bleeding *coup sur coup*. In all these cases the pulse is accelerated in proportion as the blood flows and the individual becomes enfeebled, the artery seeming fuller and harder, so that the most experienced may become embarrassed. In threatened miscarriage, wherein bleeding is often so necessary, if we practice it when the blood is red we inevitably induce abortion. I therefore always recommend those pupils who bleed for me to be guided in the quantity they draw by the colour of the fluid, and they have often been in a condition to prove the correctness of the rule. It has happened to me sometimes to close a vein immediately after having opened it, and to suddenly stop the flow upon this simple indication, and in all these cases I have never had cause to regret it. * * * * * * When a patient is about to enter upon convalescence, the pulse often becomes full and frequent, the cheeks are flushed, the eyes shining, and the skin hotter—in a word, there is fever; and it is not rare for the practitioner to hesitate whether he should abstract blood or content himself with dieting. I have always been safely guided by the colour of the blood. Black blood coagulates and reddens after it has left the vein; when it remains black on the surface it is diseased; and when it does not coagulate it is dead, as is seen in that flowing from old ulcers, uterine and other cancers. In the bleedings practised on young pregnant women red blood at first flows out and then black; the former, I believe, coming from the superficial, and the latter from the deeper-seated veins. In the treatment of a disease, then, if on opening the vein the blood is black, let it flow; if it is red, be certain that the disease is vanquished, and the point of tolerance reached. Great thirst also, which almost always follows excessive bleeding, is not a symptom to be overlooked. In organic disease the blood is generally black; but it may not always be prudent to abstract it, while if it is red we shall but increase the gravity of the disease.

Another remark, which I do not remember to have seen, is, that the blood in a diseased part may be different to that in the economy at large. I have seen it flow black from the general circulation, and red from leeches or cupping-glasses, and vice-versa. It is this that revealed to me the importance of bleeding in the treatment of cancer, or in tumours threatening to become cancerous; but that always in relation to the general vital powers; and in such a manner as to prevent general infection by absorption, which bleeding hinders or favours, according to the circumstances under which it is undertaken.

M. Tanchou goes on to state, that in the inflammatory and congestive *Diseases of the Uterus* repeated bleeding may be very useful, as long as it is not carried to excess; and that proofs of its being so are derived from the fact of the resistance of the disease, and the signs of general enfeeblement of the economy. Most of these effects of the too-great loss of blood are familiar to our readers; but he mentions two which are probably less so, viz. *constipation* and the *condition of the urine*. The stools are inodorous, infrequent, and lumpy, intestinal absorption having extracted all that was possible from them. The urine is high-coloured, but without any deposit, and emits a peculiar penetrating odour when passed, which disappears on cooling. This odour is at once recognized by the experienced observer as the most infallible sign of inanition, frequently appearing before any of the others.—*Gazette Medicale,* No. 20.

[The author, in detailing his practice still farther, recommends general bleeding in uterine affections to a far greater extent than would be countenanced in this country, and seems to much fear discontinuing it otherwise than gradually. He cautions his readers, however, against the abuse of the lancet, and not without reason, for he says, "We have seen women who have been bled 80, 95, 100 times, or even more, in one or two years!" Shade of Guy Patin arise, and testify that thy descendants are no degenerate phlebotomists! We have quoted the paper however on account of M. Tanchou's remarks on the indications derivable from the colour of the blood while flowing, which are new to us, and are so opposed to what is generally accepted, as to require confirmation.—*Rev.*]

---

### The Pneumonia of Old Persons.

Of ten cases we observed at Salpêtrière, seven were well-marked, and three doubtful. The term *doubtful* pneumonia may surprise some readers accustomed to meet with the well-known signs of the disease. But if we waited for the usual train of symptoms met with in the adult before we commenced treating pneumonia in the aged, we should often begin to act only when art had become powerless. For physicians accustomed to the aged, and who know how frequently in them it is *latent,* it suffices to observe *malaise,* fever, accelerated respiration, and a dry tongue, to direct their attention to pneumonia, especially in winter or spring-time. If we find in books that the pneumonia of the aged is more commonly than that of the adult preceded by precursory symptoms, and that it seldom shows itself in old persons suddenly, this is because too frequently the period of the commencement of pneumonia has been dated only from the appearance of stethoscopic signs. The earliest of these, and often the only one throughout the disease, is the feebleness of the respiration, or sometimes its complete absence over a certain extent. This sign, joined to bronchophony, constitutes for the experienced observer a certain indication of the presence of the disease.

The digestive and cerebral functions are much oftener disordered in the aged than in the adult. A dryness of the tongue, which alone may lead us to fear a pneumonia, is complicated with a yellow or brownish coating, bilious vomiting, and even delirium. An icteric colour of the face has not excited sufficient attention, notwithstanding its so constant co-existence with pneumonia of the apex.

Most practitioners are very circumspect in bleeding in these cases; but their fears are based upon no sound appreciation of facts. We see old pneumonic persons bled two or three times without any ill-effect, the blood retaining its normal characters and the characteristic buffing. M. Fauvel, after having bled the patient once, twice, and even three times, according to their strength and the amount of fever, and generally after having on the first day given antimony as an injection, administers from five to twelve of the following powders:—Calomel 8 grs., Opium 2 grs., Tartar emetic, 1 gr. Mix for eight Powders. Salivation does not generally take place until the 3rd or 4th day; but as soon as that is perceived, the patient's condition ameliorates; but the powders are continued until resolution has commenced. He formerly gave tartar-emetic in large doses; but was obliged to renounce it in consequence of the inflammations of the throat and bowels it gave rise to. It is to be observed, that in individuals who have long lost their teeth, salivation does not take place, or only with great difficulty, the gums remaining unaffected amidst the general buccal inflammation. In some of these patients an intestinal flux, coincident with the resolution of the pneumonia, is substituted for the salivation.—*L'Union Medicale,* No. 39.

---

### Anesthesia in Saturnine Affections.

M. Beau has remarked that all the subjects suffering from saturnine affections, manifest more or less insensibility of surface. We do not allude to that saturnine anesthesia described by authors, when it exists in a marked degree, and which is not seen above six or eight times in a hundred cases. M. Beau declares, and he has shewn us several cases now in his wards, that whatever may be the saturnine affection under which the patient is labouring, severe or slight, there is always *insensibility of the skin,* in some regions oftener than in others, and in proportion to the intensity of the saturnine disease. We saw him prick the skin of the arms and hands of several of these patients without their feeling any notable pain; so that lead may be stated to blunt sensibility. The fact is a new and important one; for it may aid in the diagnosis of some doubtful cases. —*Gazette des Hôpitaux,* No. 54.

---

### Alum as an Emteic in Croup.

Dr. Meigs, relating some cases of true croup, takes the opportunity of confirming the accuracy of the account furnished by his father long ago of the great value of alum as an emetic in this disaase. A *teaspoonful* given in honey, treacle, or other vehicle, acts more speedily and certainly than any other substance, and that without inducing prostration of the system. It is rarely necessary to repeat it.—*American Journal of the Medical Sciences,* No. 26.

---

### Cod-Liver Oil.

M. Bretonneau states that he has observed effects just as markedly beneficial to result from the use of the common whale oil as from the cod-liver oil: an important fact, seeing the great expensiveness and the frequent adulteration of the latter.

